# Caracterización molecular de pacientes con cáncer colorrectal

**DOI:** 10.7705/biomedica.5957

**Published:** 2022-05-01

**Authors:** Carlos Humberto Afanador, Katherine Andrea Palacio, Luis Fernando Isaza, Enoc Ahumada, Carlos Mauricio Ocampo, Carlos Mario Muñetón

**Affiliations:** 1 Unidad de Genética Médica, Departamento de Pediatría, Facultad de Medicina, Universidad de Antioquia, Medellín, Colombia Universidad de Antioquia Universidad de Antioquia Medellín Colombia; 2 Departamento de Cirugía, Facultad de Medicina, Universidad de Antioquia, Medellín, Colombia Universidad de Antioquia Universidad de Antioquia Medellín Colombia; 3 Hospital Fundación San Vicente de Paúl, Medellín, Colombia Hospital Fundación San Vicente de Paúl Medellín Colombia; 4 Clínica Las Vegas, Medellín, Colombia Clínica Las Vegas Clínica Las Vegas Medellín Colombia; 5 Departamento de Patología, Facultad de Medicina, Universidad de Antioquia, Medellín, Colombia Universidad de Antioquia Universidad de Antioquia Medellín Colombia

**Keywords:** neoplasias colorrectales/genética, genes supresores de tumor, oncogenes, heterogeneidad genética, inestabilidad de microsatélites, epigenómica, Colorectal neoplasms/genetics, gene, tumor suppressor, oncogenes, genetic heterogeneity, microsatellite instability, epigenomics

## Abstract

**Introducción.:**

El cáncer colorrectal tiene una alta incidencia en la población mundial. Diversas vías moleculares están involucradas en su desarrollo, entre ellas, la inestabilidad cromosómica, la inestabilidad microsatelital y la epigenética.

**Objetivo.:**

Hacer la caracterización molecular de 44 individuos con cáncer colorrectal esporádico.

**Materiales y métodos.:**

El análisis de mutaciones en los genes *APC*, *KRAS*, *TP53* y *BRAF* se hizo mediante secuenciación de Sanger; la inestabilidad microsatelital se determinó mediante electroforesis capilar utilizando cinco marcadores de repetición corta en tándem (*Short Tandem Repeat*) y el estado de metilación del promotor del gen *MLH1* se hizo con la técnica MS-PCR (*Methylation-Specific* PCR).

**Resultados.:**

La frecuencia de mutación de los genes *APC*, *KRAS* y *TP53* fue del 18,1, 25 y 4,5 %, respectivamente; las mutaciones detectadas se localizaron con mayor frecuencia en el colon derecho. La frecuencia de inestabilidad microsatelital fue del 27,2 % y el 73,1 % en los tumores con metilación en el gen *MHL1*, y el 91,6 % de los tumores con inestabilidad microsatelital presentaba metilación en el gen *MLH1*. En el grupo de tumores con estabilidad microsatelital, las mutaciones en los genes *APC*, *KRAS* y *TP53* fueron más frecuentes que en el grupo de tumores con inestabilidad microsatelital. La metilación del gen *MLH1* fue la alteración más predominante.

**Conclusiones.:**

En los pacientes con cáncer colorrectal evaluados se demostró la presencia de alteraciones moleculares en las diferentes vías genéticas, las cuales son comunes en su carcinogénesis. Los pacientes presentaron un perfil de mutaciones diferente al de otras poblaciones. Los hallazgos obtenidos en este estudio confirman la heterogeneidad molecular descrita en el desarrollo del cáncer colorrectal.

## Abstract (en)

**Introduction:**

Colorectal cancer has a high incidence in the world population. Different molecular pathways, such as chromosomal instability, microsatellite instability, and epigenetics are involved in its development.

**Objective:**

To perform molecular characterization in 44 individuals with sporadic colorectal cancer.

**Materials and methods:**

We conducted mutation analyses of the *APC*, *KRAS*, *TP53* y *BRAF* genes using Sanger sequencing techniques; microsatellite instability was determined by capillary electrophoresis with five STR genetic markers while the methylation status of the *MHL1* promotor gene was analyzed using methylation-specific PCR.

**Results:**

*APC*, *KRAS*, and *TP53* genes mutation frequency was 18.1%, 25%, and 4.5%, respectively; the somatic mutations detected were located more frequently in the right colon. The frequency of microsatellite instability was 27.2% and 73.1% of the tumors had the *MHL1* gene methylated while 91.6% of microsatellite instability-positive tumors had the methylated *MLH1* gene. The mutation profile of microsatellite stability tumors *APC*, *KRAS*, and *TP53* genes was more frequent than in the microsatellite instability-positive tumors. The methylation of the *MLH1* gene was the most predominant molecular alteration.

**Conclusions:**

We identified molecular alterations in different genetic pathways of the colorectal cancer patients evaluated, which are common in the carcinogenesis of this cancer. These patients showed a different mutational profile compared to other populations. Our findings confirm the molecular heterogeneity described in the development of colorectal cancer.

El cáncer colorrectal es una de las principales causas de muerte por cáncer en el mundo. En Colombia, es el cuarto cáncer más frecuente y el tercero en mortalidad para ambos sexos. Las variaciones geográficas del cáncer colorrectal son atribuibles a factores genéticos y ambientales, a la dieta y al estilo de vida, entre otros ^1^^-^^3^.

El 80 % de los casos de cáncer colorrectal son de tipo esporádico y el 20 % restante es de tipo familiar ^3^^,^^4^; las mutaciones germinales en los genes de reparación *MLH1* y *MSH2* originan el síndrome de Lynch (*Hereditary Nonpolyposis Colorectal Cancer*), en tanto que las del gen *APC* causan la poliposis adenomatosa familiar (*Familial Adenomatous Polyposis*); ambos síndromes corresponden al cáncer colorrectal hereditario y presentan un patrón de herencia autosómico dominante ^5^. Entre los factores de riesgo que se asocian con el desarrollo de esta neoplasia, están la dieta, la obesidad, el consumo de carnes rojas, alcohol y cigarrillo, y la diabetes de tipo 2, entre otros ^3^^,^^4^.

El cáncer colorrectal se considera una enfermedad de gran heterogeneidad genética que se origina a partir de diferentes vías genéticas y epigenéticas ^4^^-^^7^. El modelo clásico de múltiples pasos en la progresión del adenoma hacia el carcinoma, propuesto por Fearon, *et al*., involucra la inactivación de genes supresores de tumores, como el *APC*, el *TP53* y el *DCC*, y mutaciones en los oncogenes *KRAS*, *SMAD* y *BRAF*, las cuales conducen a una inestabilidad genómica; esta vía es conocida como supresora o de inestabilidad cromosómica ^8^. La vía de mutación también está involucrada en la carcinogénesis del cáncer colorrectal y se relaciona con mutaciones en los genes del sistema de reparación de bases mal apareadas (*Mismatch Repair System, MMR*) *MLH1*, *MSH2*, *MSH6*, *PMS1* y *PMS2*, los cuales inducen en los tumores con inestabilidad microsatelital ^9^. En el cáncer colorrectal esporádico, esta inestabilidad se presenta en el 15 al 20 % de los casos y ocurre principalmente por la metilación del promotor del gen *MLH1*, en tanto que, en individuos con cáncer colorrectal hereditario, como el síndrome de Lynch, se observa hasta en el 90 % de los casos ^10^^,^^11^. Otra vía implicada en el desarrollo del cáncer colorrectal es la epigenética, que se presenta por la metilación del promotor del gen *MLH1*^7^^,^^12^^,^^13^. Las vías descritas confirman la heterogeneidad genética de este cáncer, por lo que es de gran importancia determinar la clasificación molecular de los pacientes mediante oncología molecular personalizada.

Por otra parte, los estudios epidemiológicos, histopatológicos y moleculares demuestran que el espectro de alteraciones moleculares que se presenta en el colon proximal, en el distal y en el recto, son diferentes, por lo que se propone que estas alteraciones en el cáncer colorrectal varían desde el colon proximal hasta el recto ^14^^,^^15^. Asimismo, se ha demostrado que en el colon proximal son frecuentes las inestabilidades microsatelitales, las mutaciones en el gen *BRAF* y la metilación del promotor del gen *MLH1*, en tanto que, en el colon distal, son más frecuentes las mutaciones en los genes *APC* y *TP53* y poco frecuentes las inestabilidades microsatelitales ^15^. Por el contrario, en el cáncer de recto no es común observar inestabilidades microsatelitales, metilación de *MLH1* ni mutaciones en el *BRAF*^14^^,^^15^.

Dada la complejidad y heterogeneidad del cáncer colorrectal, en diversos estudios se subraya la importancia de identificar subgrupos moleculares de esta neoplasia con base en la detección de alteraciones moleculares en las vías descritas. Recientemente, un consorcio internacional (*Centers for Medicare and Medicaid Services*) propuso un consenso molecular de subtipos de cáncer colorrectal, con una clasificación molecular más detallada y completa a partir de la integración de la información obtenida de los análisis de mutaciones, los cambios en el número de copias, el patrón de metilación, la expresión del microARN y la proteómica de 4.000 pacientes, cuyo resultado final definió un patrón de cuatro subtipos moleculares (CMS 1 a 4) ^16^. Este consorcio, además de describir las características genéticas de los tumores en el cáncer colorrectal, también las correlacionó con los datos clínico-patológicos de los pacientes y con el pronóstico de la enfermedad. Cabe mencionar que la clasificación propuesta significó un gran avance en su caracterización molecular.

Además, la caracterización molecular de los pacientes con cáncer colorrectal es de gran utilidad en el manejo clínico de la enfermedad, ya que algunas de las alteraciones moleculares descritas proporcionan información importante sobre la reacción de los pacientes a determinados tratamientos antineoplásicos, lo que permite la selección de la terapia más eficaz para cada individuo y podría aumentar su tasa de supervivencia. Es el caso específico del gen *KRAS*, este se considera un biomarcador importante para predecir la reacción a terapias anti-*EGFR*. Los pacientes con cáncer colorrectal metastásico sin mutaciones en el *KRAS*, tienen una mejor respuesta terapéutica con anticuerpos monoclonales anti-*EGFR*, como el panitumumab o cetuximab, en tanto que los pacientes que presentan un *KRAS* mutado son resistentes a estas terapias ^4^^,^^17^^,^^18^.

En este mismo sentido, la detección de la mutación *V600E* en el gen *BRAF* es también clave para predecir la respuesta a terapias con anticuerpos monoclonales, ya que los pacientes con esta mutación no tienen una buena respuesta a este tipo de tratamiento ^4^^,^^18^^,^^19^. Como ocurre con las mutaciones en los genes *KRAS* y *BRAF*, la determinación de la inestabilidad microsatelital en la respuesta terapéutica también es clínicamente importante porque los pacientes con este tipo de inestabilidad tienen un mejor pronóstico de supervivencia que quienes no la muestran lo son, es decir, son estables ^4^. Además, es útil para confirmar el diagnóstico molecular de pacientes y familias con sospecha de síndrome de Lynch o cáncer colorrectal no polipósico hereditario. Los hallazgos de estudios clínicos también informan que estos pacientes no mejoran con la terapia con 5-fluorouracilo, pero sí tienen una mejor respuesta terapéutica con oxiplatino ^19^^,^^20^. Por ello, se recomienda incluir el análisis de inestabilidad microsatelital en el diagnóstico molecular de pacientes con cáncer colorrectal.

El objetivo de nuestro estudio fue analizar mutaciones somáticas en los genes *APC*, *KRAS*, *BRAF* y *TP53*, y determinar la inestabilidad microsatelital y el patrón de metilación del gen *MLH1* en 44 pacientes colombianos con cáncer colorrectal esporádico, así como correlacionar los datos clínico-patológicos con los hallazgos moleculares.

## Materiales y métodos

### Pacientes y muestras

Se hizo un estudio descriptivo de corte transversal. La población de estudio estuvo constituida por 44 pacientes, 27 mujeres y 17 hombres, con diagnóstico de cáncer colorrectal esporádico, procedentes del departamento de Antioquia, y con una media de edad de 60,5 años (rango: 12 a 90 años). Ningún paciente tenía historia familiar o personal de cáncer o tratamiento antineoplásico antes de la cirugía. Todos los estudios histopatológicos fueron revisados y confirmados por un patólogo experto. A cada paciente se le solicitó la participación voluntaria en el estudio y la firma del consentimiento informado.

Los cirujanos recolectaron muestras del tejido del tumor primario y del tejido normal adyacente simultáneamente en cada caso, mediante resección quirúrgica en el Hospital Universitario San Vicente Fundación y la Clínica León XIII de Medellín entre el 2015 y el 2016. Cada muestra tumoral se dividió en dos porciones, una para el estudio histopatológico a cargo de un patólogo experto, y la otra se almacenó a -80 °C para el posterior análisis molecular. La información personal y de los diagnósticos histopatológicos se obtuvo de las historias clínicas de los pacientes.

### Análisis molecular

*Extracción de ADN*. El ADN genómico se extrajo a partir de tejido tumoral y tejido normal de cada paciente, utilizando el estuche comercial QIAamp DNA Mini Kit™ (Qiagen, Hilden Germany) y siguiendo las recomendaciones del fabricante. El ADN se cuantificó en un espectrofotómetro NanoDrop 2000c Spectrophotometer™ (Thermo Scientific, USA) y, una vez extraído, se diluyó y se almacenó a -20 °C.

*Amplificación del ADN y análisis de mutaciones en los genes APC, KRAS, BRAF y TP53*. El ADN extraído del tejido tumoral se amplificó por PCR en un termociclador Gene Amp PCR System 9700™ (Applied Biosystems, USA), utilizando cebadores específicos para cada gen. El análisis de mutaciones de los genes se realizó amplificando las regiones que son puntos clave (hotspot) para mutaciones somáticas en los genes *APC* (exón 15; región MCR), *KRAS* (exones 2 y 3), y *TP53* (exones 5-8). A todos los tumores con inestabilidad microsatelital positiva se les hizo el análisis de la mutación V600E en el exón 15 del gen *BRAF*, utilizando cebadores específicos para esta región. La reacción de la PCR se hizo en un volumen final de 35 μl que contenía 50 ng de ADN tumoral con las siguientes concentraciones: 1X de solución tampón de reacción 10X, 1,05 mM de MgCl_2_, 2 μM de dNTP, 0,4 μM de cada iniciador y 1,4 U de Taq ADN polimerasa (Invitrogen, Brasil). Los productos amplificados por la PCR se analizaron mediante electroforesis en geles de agarosa al 2 %, teñidos con 2 μl de GelRed, y se almacenaron a -20 °C hasta el momento de la secuenciación.

*Análisis de inestabilidad microsatelital*. La inestabilidad microsatelital se analizó en el ADN extraído del tejido tumoral y del normal de cada paciente, y se evaluó con la amplificación por PCR de un panel de cinco marcadores STR previamente descritos (Bat-25, Bat-26, NR-21, NR-27 y NR-24) ^11^. Los tamaños alélicos del ADN tumoral y del normal se compararon y se analizaron mediante electroforesis capilar en un analizador genético ABI 3770. La inestabilidad microsatelital se determinó cuando se detectaron alelos en la muestra del ADN tumoral que no estaban presentes en la muestra del normal. Se definió como inestabilidad microsatelital alta (MSI-H) la presencia de dos o más marcadores inestables en el tumor; como inestabilidad microsatelital baja (MSI-L), la de un solo marcador inestable, y la estabilidad microsatelital, cuando no se detectaba ningún marcador inestable.

*Análisis de metilación en el gen MLH1 por MS-PCR*. El ADN tumoral se modificó con bisulfito de sodio, usando el estuche comercial EZ- DNA Methylation Direct Kit™ de Zymo Research y siguiendo las recomendaciones del fabricante. El ADN modificado se almacenó a -20 °C. La metilación del promotor del gen *MLH1* se hizo con la técnica MS-PCR. El ADN tumoral modificado con bisulfito de sodio se amplificó por PCR utilizando iniciadores específicos para regiones metiladas y no metiladas del promotor del gen *MLH1* en un termociclador Gene Amp PCR System 9700™ (Applied Biosystems, USA). La MS-PCR se hizo en un volumen final de 10 μΙ que contenía 2 μl de ADN modificado, solución tampón 1X, 1,5 μM de MgCl_2_, 0,2 mm de dNTP, 0,4 μΜ de cada cebador y 0,8 μΙ de Taq polimerasa. Los productos amplificados de la MS-PCR se corrieron en electroforesis en geles de agarosa al 2 % teñidos con GelRed.

*Secuenciación*. Todos los productos de las PCR amplificados se purificaron y secuenciaron directamente en ambas cadenas con el método Sanger. La secuenciación se hizo en un analizador genético ABI 3730xl DNA Analyzer Applied Biosystems™. Las secuencias obtenidas se editaron y analizaron con el programa Chromas Pro y se alinearon con las secuencias de referencia publicadas en el GenBank (NCBI), cuyos códigos de acceso son NT_034772.6 (APC), NT_009714.17 (KRAS), NT_010718.16 (TP53) y NC_000007.14 (BRAF). El análisis bioinformático de las variantes génicas detectadas también se realizó con las bases de datos del NCBI dbSNP (http://www.ncbi.nlm.nih.gov/snp), 1000 genomas y COSMIC.

### Análisis estadísticos

Los resultados se analizaron mediante el programa SPSS™, versión 23 (IBM Corp.). Con las pruebas de ji al cuadrado o con la corrección de continuidad de Yates y la prueba exacta de Fisher se exploró la asociación entre las mutaciones, los polimorfismos, la inestabilidad microsatelital y la metilación, y el diagnóstico histopatológico, la edad y el sexo. Todos los valores de p calculados fueron bilaterales y los p<0,05 se consideraron estadísticamente significativos.

El protocolo del estudio fue aprobado por el Comité de Bioética para experimentación en humanos de la Universidad de Antioquia.

## Resultados

Se estudiaron 44 pacientes con cáncer colorrectal esporádico; la edad promedio fue de 60,5 años, con un rango entre los 12 y los 90 años. El 61,3 % (27/44) de los pacientes correspondía a mujeres y el 38,6 % (17/44), a hombres. El 81,8 % (36/44) tenía cáncer de colon y, el 11,3 % (5/44), de recto. Los datos de edad y sexo, y los hallazgos histopatológicos y moleculares de los pacientes analizados, se presentan en el cuadro 1.

**Cuadro 1:** Descripción general de edad, sexo y los hallazgos histopatológicos y moleculares en los 44 pacientes con cáncer colorrectal estudiados

		n (%)	APC n (%)		KRAS n (%)		TP53 n (%)		MSI n (%)		
			Normal	mutado	Normal	mutado	Normal	mutado	MSI-H	MSI-L	MSS	
Número de pacientes		44	36 (81,8)	8 (18,1)	33 (75)	11 (25)	42 (95,4)	2 (4,5)	5 (11,3)	7 (15,9)	32 (72,7)
Edad media		60,5	57	58,5	57	47,5	57	62,5	68	57,1	61
Sexo											
	Masculino	17 (38,6)	15 (34)	2 (4,5)	13 (29,5)	4 (9)	17 (38,6)	-	4 (9)	4 (9)	9 (20,4)	
	Femenino	27 (61,3)	21 (47,7)	6 (13,6)	20 (45,4)	7 (15,9)	25 (56,8)	2 (4,5)	1 (2,2)	3 (6,8)	23 (52,2)
Estadio											
	I	8 (21,1)	7 (15,9)	1 (2,2)	7 (15,9)	1 (2,2)	8 (18,1)	-	1 (2,2)	2 (4,5)	5 (11,3)	
	II	13 (34,2)	11 (25)	2 (4,5)	12 (27,2)	1 (2,2)	11 (25)	2 (4,5)	3 (6,8)	1 (2,2)	9 (20,4)	
	III	6 (15,8)	5 (11,3)	1 (2,2)	5 (11,3)	1 (2,2)	6 (13,6)	-	-	-	6 (13,6)	
	IV	11 (28,9)	7 (15,9)	4 (9)	5 (11,3)	6 (13,6)	11 (25)	-	-	2 (4,5)	9 (20,4)
Tipo histológico											
	Bien diferenciado	24 (54,4)	20 (45,4)	5 (11,3)	18 (40,9)	7 (15,9)	23 (52,2)	2 (4,5)	3 (6,8)	4 (9)	18 (40,9)
	Moderadamente diferenciado	10 (22,7)	9 (20,4)	1 (2,2)	10 (22,7)	-	10 (22,7)	-	1 (2,2)	1 (2,2)	8 (18,1)
	Mucinoso	5 (11,3)	3 (6,8)	1 (2,2)	2 (4,5)	3 (6,8)	5 (11,3)	-	1 (2,2)	-	4 (9)
	Sarcoma miofibroblástico	1 (2,2)	-	1 (2,2)	-	-	-	-	-	-	-
Localización del tumor											
	Colon ascendente	16 (36)	11 (25)	5 (11,3)	11 (25)	5 (11,3)	15 (34,0)	1 (2,2)	2 (4,5)	3 (6,8)	11 (25)	
	Colon transverso	5 (11,3)	4 (9)	1 (2,2)	2 (4,5)	3 (6,8)	5 (11,3)	-	1 (2,2)	1 (2,2)	3 (6,8)	
	Colon descendente	15 (34)	14 (40,9)	1 (2,2)	13 (38,6)	2 (6,8)	15 (43,1)	-	2 (4,5)	2 (4,5)	11 (36,3)	
	Recto	5 (11,3)	4 (9)	1 (2,2)	4 (9)	1 (2,2)	4 (9)	1 (2,2)	-	-	5 (11,3)

La frecuencia de mutaciones en las 44 muestras analizadas fue del 47,7 % (21/44). Todas las mutaciones identificadas fueron por sustitución de bases. De estas, el 23,8 % (5/21) sin sentido y el 76,2 % (16/21) de sentido erróneo (cuadro 2). Las mutaciones detectadas fueron más frecuentes en mujeres (15/21) que en hombres (6/21) y se encontraron con mayor frecuencia en el colon ascendente, con el 52,3 % de los casos (11/21), seguido del colon transverso, con el 19 % (4/21), en tanto que el colon descendente y el recto presentaron la misma frecuencia de mutaciones, con el 14,2 % (3/21). Además, la frecuencia de polimorfismos en los genes evaluados fue alta; se identificaron en total cinco polimorfismos diferentes en las 44 muestras analizadas. El más frecuente fue el rs41115 (c.4479 G>A, p.Thr1493Thr), ubicado en el exón 15 del gen *APC* en el 75 % de las muestras (cuadro 3). Las mutaciones y los polimorfismos identificados en este estudio están reportados en las bases de datos COSMIC, SNP en NCBI y 1000 genomas.

**Cuadro 2:** Mutaciones identificadas en los genes *APC*, *KRAS* y *TP53* en los 44 pacientes con cáncer colorrectal evaluados

Gen	n	Sexo	Posición	Codón	Mutación	Cambio en aminoácidos	Tipo de cambio	Consecuencia
*APC*	1	F	c.4420A>G	1474	ACT >GCT	p.A1474T	Sustitución	Cambio de sentido
	3	F	c.4463 T>C	1488	TTA >TCA	p.L1488S	Sustitución	Cambio de sentido
	1	F	c.4562 G>T	1521	GAA >TAA	p.E1521*	Sustitución	Sin sentido
	1	F	c.4651A>T	1551	AAA>TAA	p.K1551*	Sustitución	Sin sentido
	2	M	c.4348C>T	1450	CGA>TGA	p.R1450*	Sustitución	Sin sentido
*KRAS*	6	4 F 2 M	c.35G>A	12	GGT>GAT	P.G12D	Sustitución	Cambio de sentido
	2	1 F 1 M	c.34G>A	12	GGT>AGT	P.G12S	Sustitución	Cambio de sentido
	3	2 F 1 M	c.38G>A	13	GGC>GAC	P.G13D	Sustitución	Cambio de sentido
*TP53*	1	F	c.493C>T	165	CAG>TAG	p.Q165*	Sustitución	Sin sentido
	1	F	c.546C>A	182	TGC>TGA	P.C182*	Sustitución	Sin sentido

* Representa el codón de parada

**Cuadro 3:** Polimorfismos identificados en los genes APC, KRAS y TP53 en los 44 individuos con cáncer colorrectal analizados

GEN	n	%	Exón / Intrón	Posición c.ADN	Cambio en aminoácidos	SNP
*APC*	33	75	Exón 15	c.4479G>A	T1493T	rs41115
	1	2,3	Exón 15	c.4326 T>A	P1442P	rs67622085
*KRAS*	8	18,2	Intrón 2	c.111+190A>T	NA	rs12228277
*TP53*	9	20,5	Intrón 7	c.782+72C>T	NA	rs12947788
	9	20,5	Intrón 7	c.782+92T>G	NA	rs12951053

NA: no aplica

**Figura 1:**
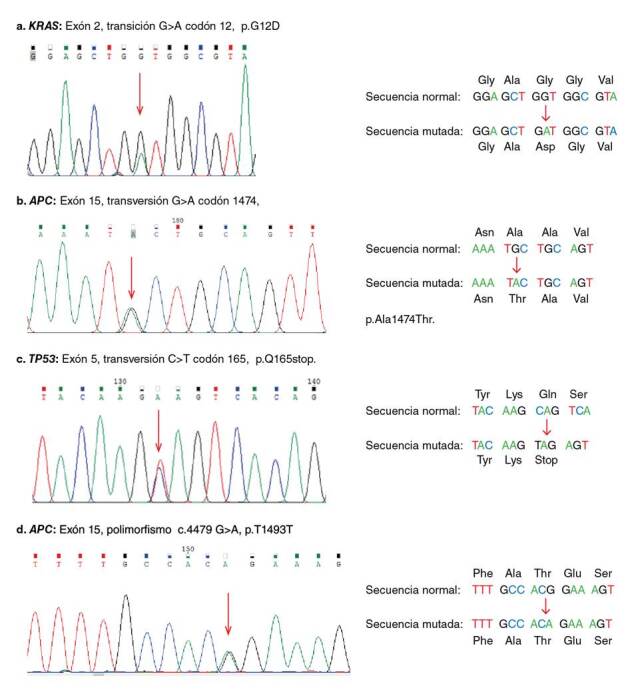
Cromatogramas obtenidos de la secuenciación directa que muestran diferentes mutaciones identificadas en los genes analizados en las muestras de cáncer colorrectal. A. Mutación en *KRAS*, codón 12 G>A. B. Mutación en *APC*, codón 1474 G>A. C. Mutación en *TP53*, codón 165 C>T. D. Polimorfismo en APC c.4479 G>A, p.T1493T. Las flechas muestran el sitio del cambio del nucleótido.

*KRAS* fue el gen más frecuentemente mutado, con el 25 % (11/44). Las mutaciones encontradas fueron más comunes en mujeres (7/11) que en hombres (4/11). Las mutaciones puntuales fueron más frecuentes en el codón 12 (72,7 %), que en el codón 13 (27,3 %) del exón 2; todas las mutaciones detectadas fueron del tipo de transiciones (cuadro 2). La mutación por sustitución más común (54,5 %) en el gen *KRAS* fue la c.35G>A p.G12D en el codón 12 (figura 1), seguida de la mutación c.38G>A, p.G13D en el codón 13, con el 27,3 %. Las mutaciones detectadas en este gen generaron un cambio de sentido en la secuencia de aminoácidos de la proteína y el 11,3 % se localizó en el colon ascendente, el 6,8 % en el colon transverso y descendente, y el 2,2 % en el recto. Se observó que el 13,6 % de las muestras de tumores con *KRAS* mutado estaba en estadio IV. Por otra parte, además de las secuencias obtenidas para este gen, también se identificó en el 18,2 % (8/44) de las muestras un polimorfismo en el intrón 2, el rs12228277 (c.111+190 A > T) (cuadro 3). Por el contrario, en el análisis de las secuencias en el codón 61 del exón 3 del *KRAS*, no se identificaron mutaciones o polimorfismos, como tampoco se encontró la mutación V600E del gen *BRAF* en las muestras de tumores con *KRAS* mutado.

Por otro lado, la frecuencia de mutación del gen *APC* en la región MCR del exón 15 fue del 18,1 % (8/44). Las mutaciones identificadas fueron más comunes en mujeres (6/8) que en hombres (2/8). La mutación más común fue la c.4463 T>C, p.L1488S en el codón 1488, con el 37,5 % (cuadro 2). En este gen, también se identificaron dos polimorfismos, el rs41115 (c.4479 G>A, p.Thr1493Thr) en el 75 % (33/44) de los casos y el rs67622085 (c. 4326 T>A, p.Pro1442Pro) en un solo caso (cuadro 3, figura 1). Las mutaciones se identificaron con mayor frecuencia en el colon ascendente, con el 11,3 % (5/44), en tanto que en el colon transverso, el descendente y el recto, se identificó una sola mutación en cada uno de ellos. De las ocho mutaciones puntuales identificadas en *APC*, tres eran de transición y dos de transversión; dos mutaciones correspondían a cambio de sentido y las otras tres fueron sin sentido y generaron una proteína truncada (cuadro 2, figura 1).

En cuanto al gen *TP53*, solo se identificaron dos mutaciones en todas las muestras analizadas, correspondientes al 4,5 %; las mutaciones se localizaron en el exón 5, una en el codón 165, de tipo transición, y la otra en el codón 182, de tipo transversión, las cuales generan un codón de parada prematura (cuadro 2, figura 1). Las dos mutaciones identificadas en muestras de mujeres se localizaron en el colon ascendente y el recto. De forma similar a lo encontrado para los genes *APC* y *KRAS*, en el *TP53* se detectaron dos polimorfismos en el intrón 7, el rs12947788 (c.782+72C > T) y el rs12951053 (c.782+92T > G), con cosegregación en el 20,5 % (9/44) de las muestras analizadas (cuadro 3). Por otra parte, en ninguna de las 44 muestras se detectaron mutaciones simultáneas en los genes analizados; solo el 9 % (4/44) de ellas tenía mutaciones concomitantes en los genes APC y KRAS.

**Figura 2:**
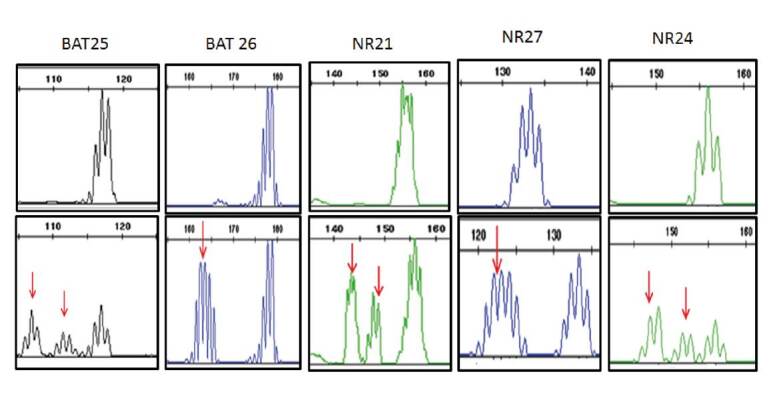
Imagen de la electroforesis capilar de una muestra de cáncer colorrectal con MSI-H. En la parte superior, se observa el perfil de los cinco marcadores STR (*Short Tandem Repeat*) del tejido normal. En la parte inferior, se observa el perfil de los marcadores STR del tejido tumoral del mismo caso que evidencia una inestabilidad microsatelital positiva alta en todos los marcadores evaluados. Las flechas indican la inestabilidad detectada con cada uno de los marcadores utilizados.

Por otra parte, la inestabilidad microsatelital se encontró en el 27,2 % (12/44) de las muestras de tumores analizadas. La MSI-H se determinó en el 11,3 % (5/44) y, la MSI-L, en el 15,9 % (7/44), en tanto que el 72,7 % (32/44) hubo estabilidad microsatelital (cuadro 1, figura 2). La inestabilidad microsatelital fue más común en hombres (8/44) que en mujeres (4/44), con el 18 y el 9 %, respectivamente, y una diferencia estadísticamente significativa (p=0,047). El promedio de edad de los pacientes con inestabilidad microsatelital fue de 60,5 años. La distribución de la inestabilidad microsatelital fue de 11,3 % (5/44) en el colon ascendente, 4,5 % (2/44) en el transverso y de 11,3 % (5/44) en el descendente (cuadro 1). No se detectó inestabilidad microsatelital en las cinco muestras de recto. Se observó que el 15,9 % de las muestras con inestabilidad microsatelital positiva estaban en los estadios I y II. Por otro lado, en el análisis de mutaciones realizado en las 12 muestras de tumores con inestabilidad microsatelital positiva y en un grupo de tumores con estabilidad microsatelital, no se identificó la mutación V600E del gen *BRAF*.

El estado de metilación del promotor del gen *MLH1* se determinó en 41 de 44 muestras. La frecuencia de metilación fue del 73,1 % (30/41). De las 30 muestras con metilación del *MLH1*, el 63,3 % (19/30) procedía de mujeres con una edad promedio de 62,8 años y, el 36,6 % restante (11/30), de hombres con un promedio de edad de 63,6 años. La metilación del gen *MLH1* fue más frecuente en el colon ascendente, con 36,6 % (11/30), seguido del descendente, con 33,3 % (10/30), y el transverso y el recto, con 13,3 % (4/30) y 10 % (3/30), respectivamente. En cuanto al estado de metilación y al estadio del tumor, se observó una frecuencia del 46,6 % de metilación en los estadios I y II, en tanto que, en los estadios III y IV, fue del 40 %.

**Cuadro 4:** Descripción general de las características patológicas de las 12 muestras de cáncer colorrectal positivas para MSI y la relación con los hallazgos de mutaciones en los genes analizados y los de metilación en el gen *MLH1*

Caso	Edad	Sexo	Localización	Estadio	APC	KRAS	TP53	MSI	MLH1
1	70	F	Ascendente	IV				MSI-L	M
2	ND	F	ND*	ND				MSI-L	M
3	72	M	Ascendente	II				MSI-H	M
4	90	F	Transverso	IV				MSI-L	M
5	58	M	Descendente	I				MSI-H	M
6	81	M	Ascendente	II				MSI-H	M
7	36	F	Transverso	ND		c.35G>A		MSI-H	M
8	62	M	Ascendente	I				MSI-L	M
9	81	M	Descendente	I				MSI-L	M
10	53	M	Descendente	II				MSI-H	M
11	79	M	Descendente	II				MSI-L	M
12	ND	M	Ascendente	ND		c.35G>A		MSI-L	UM

ND: no determinado; M: con metilación; UM: sin metilación

Asimismo, el 91,6 % (11/12) de las muestras de tumores positivas para inestabilidad microsatelital presentaba metilación en el gen *MLH* y no portaba la mutación V600E del gen *BRAF*. En cuanto al estado de metilación, la inestabilidad microsatelital y el perfil de mutaciones en los genes analizados en este grupo de tumores, se observaron simultáneamente con mayor frecuencia en hombres que en mujeres en el colon ascendente, con 41,6 % (5/12) y en el estadio II, con 33,3 % (4/12). Solo dos muestras (18,2 %) tenían mutaciones en el gen *KRAS* (c.35G>A p.G12D) (cuadro 4).

Con respecto a las 32 muestras de tumores estabilidad microsatelital, estas fueron más frecuentes en mujeres, con el 71,8 % (23/32), y a una edad promedio de 61 años, y se encontraron con mayor frecuencia en el colon descendente y el recto, con el 34,3 % (11/32) y el 15,6 % (5/32), respectivamente. La estabilidad microsatelital en nuestras muestras fue más frecuente en la parte distal del colon y en los estadios II y IV, con el 56,2 % (18/32). En este grupo de tumores con estabilidad microsatelital, la metilación del gen *MLH1* fue del 59,3 % (19/32) y la frecuencia de mutaciones en los genes *APC*, *KRAS* y *TP53* fue del 59,3 % (19/32). De este último porcentaje, el 47,5 % tenía mutaciones en el KRAS, 42,1 % en el *APC* y 10,5 % en el *TP53*, lo que es similar a lo encontrado en el grupo de tumores con inestabilidad microsatelital, en el que el KRAS fue el gen más frecuentemente mutado en las muestras con estabilidad microsatelital. Solo tres muestras de este grupo de tumores tenían mutaciones en los genes *APC* y *KRAS* simultáneamente.

## Discusión

El desarrollo del cáncer colorrectal se caracteriza por la acumulación de múltiples alteraciones moleculares en diferentes vías genéticas como la inestabilidad cromosómica, la inestabilidad microsatelital y la epigenética, las cuales afectan la expresión de los genes supresores de tumores, los protooncogenes y los genes de reparación.

En el presente estudio, se llevó a cabo la caracterización molecular de 44 pacientes colombianos con cáncer colorrectal esporádico, sabiendo que las mutaciones en los genes *APC*, *KRAS* y *TP53* son esenciales en la transformación y progresión de adenoma a carcinoma colorrectal. Se encontró una frecuencia total de mutaciones en estos tres genes del 47,7 %.

La frecuencia de mutación del gen *APC* en nuestro estudio fue menor comparada con la reportada en otros estudios (21,22), en los que se informan frecuencias de mutaciones de hasta el 70 % al analizar las secuencias de todos los exones que contienen el gen *APC*. Nuestro estudio se restringió al análisis de mutaciones en la región *MCR*, que representa cerca del 10 % de toda la región codificadora de *APC* y se considera un punto clave (*hotspot*) para mutaciones en este gen ^22^.

La poca frecuencia de mutaciones en el *APC* en estos pacientes colombianos con cáncer colorrectal corrobora los resultados previamente reportados por nosotros en otra cohorte de pacientes ^23^. Sin embargo, nuestro resultado es similar a los reportados por Al-Shamsi, *et al*., y Syed, *et al*., quienes informan bajas frecuencias de mutaciones en el gen APC en poblaciones del sur de Asia y la península arábiga, con el 27,3 y el 12,8 %, respectivamente ^24^^,^^25^. No obstante, es necesario aumentar el número de pacientes analizados para validar nuestros resultados en este gen y evaluar todos sus exones. Las mutaciones puntuales más frecuentes en los pacientes con cáncer colorrectal fueron la c.4463 T>C p.L1488S, seguida de la c.4348C>T p.R1450*, lo que coincide con lo reportado en otros estudios de cáncer colorrectal esporádico ^22^^,^^23^. Las diferencias encontradas entre nuestros resultados y los reportados por otros autores, además del tamaño muestral, permiten sugerir que las frecuencias y el patrón de mutaciones somáticas en el gen *APC* en el cáncer colorrectal difieren entre poblaciones, posiblemente por la variación geográfica de las frecuencias de las mutaciones y por factores étnicos.

Las mutaciones en el gen supresor de tumores *APC* se consideran un evento inicial crítico en la carcinogénesis del cáncer colorrectal esporádico, puesto que las mutaciones en la región MCR afectan el dominio funcional de unión de la proteína β-catenina, la cual se acumula en el citoplasma de las células colorrectales y, por esta vía, se promueve la formación del cáncer ^8^^,^^18^^,^^22^^,^^25^.

Debe tenerse en cuenta que en el *APC* ocurren otros tipos de alteraciones genéticas, como la pérdida de hetorocigocidad hasta en el 30 %, deleciones cromosómicas en la región 5q21 y la hipermetilación del promotor del gen *APC* (entre 30 y 45 %) (8,25-27). Por lo tanto, se sugiere que en las muestras tumorales analizadas en este estudio no se podría excluir la presencia de las anteriores alteraciones.

En el presente estudio, el *KRAS* fue el gen más frecuentemente mutado, con una frecuencia de mutación similar a la reportada en otros estudios y un rango entre el 13 y el 66 % en pacientes con cáncer colorrectal esporádico de diferentes poblaciones ^17^^,^^19^^,^^21^^,^^22^. En países como China, Estados Unidos y Francia, se reportan las frecuencias más altas de mutaciones (40 a 66 %), en tanto que, en algunas poblaciones asiáticas, se informan bajas frecuencias, de 13 a 26,5 %, similar a lo encontrado en nuestro trabajo ^19^^,^^24^^,^^28^^,^^29^. Asimismo, nuestros resultados concuerdan con la frecuencia de mutaciones en este gen previamente reportada en otro estudio en pacientes con cáncer colorrectal ^23^.

Las mutaciones puntuales identificadas en el *KRAS* fueron más comunes en mujeres que en hombres, y con mayor prevalencia en el codón 12 que en el 13, en tanto que en el codón 61 no se detectaron mutaciones. Nuestros resultados concuerdan con lo reportado en otros estudios en que se informa que hasta el 97 % de las mutaciones ocurre en los codones 12 y 13 del exón 2 ^28^^,^^29^. La mutación por sustitución de bases más predominante en las muestras tumorales analizadas fue la G12D, similar a lo reportado en otros estudios ^17^^,^^19^^,^^22^^,^^28^. Esta mutación representa más del 50 % del total de mutaciones reportadas en el *KRAS* en pacientes con cáncer colorrectal, por lo que se considera que es clave en su carcinogénesis. En este estudio, también se detectaron otras mutaciones frecuentes en el *KRAS* como la G13D y la G12S. Sin embargo, no se detectó la mutación G12V, la cual es frecuente en otras poblaciones ^19^^,^^22^^,^^24^^,^^28^. Las mutaciones detectadas en el *KRAS* fueron más comunes en el colon ascendente que en el colon descendente y el recto, resultados que coinciden con los reportados por otros autores ^17^^,^^19^^,^^22^^,^^29^.

En el presente estudio, los casos de cáncer colorrectal con el *KRAS* mutado no tenían la mutación V600E del gen *BRAF*, resultados que corroboran lo reportado en varios estudios que evidencian que las mutaciones en los genes *KRAS* y *BRAF* son mutuamente excluyentes ^19^^,^^22^^,^^28^^,^^30^. En general, en el cáncer colorrectal esporádico se reporta una baja frecuencia (0 a 12 %) de mutaciones en el gen *BRAF*, y nuestros resultados están dentro de este rango ^4^^,^^18^^,^^19^^,^^24^.

Las mutaciones puntuales en el gen *KRAS* originan una activación constitutiva de este oncogén, lo que produce una desregulación en la vía de señalización RAS/RAF/MAPK, la cual estimula la proliferación celular, un evento clave en el proceso de transformación de adenoma a carcinoma en el cáncer colorrectal ^4^^,^^8^^,^^18^. Asimismo, las mutaciones en el *KRAS* se asocian con un mal pronóstico de la enfermedad y, en estudios clínicos de pacientes con cáncer colorrectal metastásico y KRAS mutado, se informa que estos pacientes no tienen una respuesta eficaz a la terapia con anticuerpos monoclonales anti-EGFR (I-EGFR) ^17^^,^^18^^,^^19^^,^^29^^,^^30^. Por ello, es importante hacer la caracterización molecular del *KRAS* en los pacientes con cáncer colorrectal esporádico, pues permite la identificación de subgrupos moleculares.

En cuanto al gen *TP53*, en este estudio se encontró una baja frecuencia (4,5 %) de mutaciones, lo que difiere significativamente de lo reportado por varios autores en pacientes con cáncer colorrectal de diferentes poblaciones, con hasta el 60 % de mutaciones cuando se evalúan todos los exones del gen ^21^^,^^22^^,^^24^^,^^31^^,^^32^. Este estudio se enfocó en el análisis molecular de los exones 5 al 8, en los cuales se reportan las mayores frecuencias de mutaciones en el TP53 (*hot-spot mutation*) ^18^^,^^22^^,^^33^. Las dos mutaciones puntuales identificadas en el *TP53* en nuestro estudio se detectaron en los codones 165 y 182, y están entre las mutaciones con menor frecuencia reportadas en diferentes poblaciones. Las mutaciones con mayor frecuencia en el *TP53* se han reportado en los codones 175, 245, 248, 273 y 282 ^22^^,^^24^^,^^33^^,^^34^. Sin embargo, nuestros resultados corroboran los obtenidos por nuestro grupo en un estudio previo en pacientes con cáncer colorrectal ^23^, pues en los dos estudios se reportaron bajas frecuencias de mutaciones en el gen *TP53*. A partir de estos hallazgos, se sugiere que los pacientes con cáncer colorrectal esporádico evaluados presentan un perfil de mutaciones en el *TP53* de frecuencia y tipo diferentes a los informados en otras poblaciones. Las diferencias entre nuestros resultados y los de los otros estudios podrían explicarse por el tamaño de la muestra, la exposición exógena a químicos, los hábitos, el estilo de vida y la etnia.

Contrariamente a la baja frecuencia de mutaciones puntuales detectada en el *TP53* en el presente estudio, en otros estudios previos se han reportado altas frecuencias de aneuploidías del cromosoma 17 y deleciones en el locus 17p13.1, en el que se localiza el gen *TP53*^35^^,^^36^. Por ello, en las muestras tumorales de cáncer colorrectal evaluadas en el presente estudio, no se podría excluir la presencia de estas alteraciones de tipo cromosómicas.

En este estudio, solo se encontraron mutaciones concomitantes en los genes *APC* y *KRAS*; no se identificaron mutaciones simultáneas en *APC*, *KRAS* y *TP53*, hallazgos que concuerdan con lo reportado para el cáncer colorrectal esporádico por otros autores ^22^^,^^23^^,^^24^^,^^31^. Por lo tanto, se considera que las mutaciones simultáneas en estos tres genes son un evento infrecuente en la carcinogénesis de esta neoplasia y que, a pesar del modelo genético de múltiples pasos propuesto por Fearon, *et al*., para la misma ^8^, no todos los casos se ajustan a él. En este sentido, se sugiere que las mutaciones en estos genes ocurren por vías moleculares independientes durante el desarrollo del cáncer colorrectal ^23^^,^^24^.

Por otra parte, los análisis de mutación en los genes evaluados permitieron la identificación de varios polimorfismos; se resalta particularmente la identificación del polimorfismo c.4479G>A p.T1493T en el exón 15 del gen *APC*, con una frecuencia del 75 % en las muestras de cáncer colorrectal esporádico analizadas. Este hallazgo corrobora resultados previos reportados por primera vez por nuestro grupo en pacientes colombianos con cáncer colorrectal y de estómago ^23^. La asociación de este polimorfismo y el riesgo de desarrollar cáncer colorrectal no está bien esclarecida. En algunos estudios, se ha encontrado dicha asociación, pero en otros no ^37^^-^^39^. Este polimorfismo está reportado en la base de datos del proyecto de 1.000 genomas, fase 3, en población antioqueña. Por lo tanto, se requieren más estudios en la población colombiana para establecer su asociación con el riesgo de desarrollar cáncer colorrectal.

En resumen, los resultados del análisis de mutaciones en las muestras de cáncer colorrectal analizadas sugieren un perfil de mutaciones particular, caracterizado por bajas frecuencias de mutación en los genes *APC*, *KRAS*, *BRAF* y *TP53*, en comparación con las informadas en otras poblaciones. Por ello, se necesitan más estudios con muestras de mayor tamaño para validar los resultados reportados aquí, así como estudios genómicos con técnicas de secuenciación de nueva generación para analizar el genoma tumoral completo y obtener una mejor caracterización genómica del cáncer colorrectal. Además de por el tamaño de la muestra, el perfil de mutaciones particular observado en el presente estudio podría estar influenciado por diferentes factores, como el estilo de vida, la dieta, el tabaquismo, la raza y otros aspectos genéticos.

La frecuencia de la inestabilidad microsatelital (27,2 %) en las muestras analizadas de tumores de cáncer colorrectal, concuerda con las informadas en otros estudios. El análisis de inestabilidad microsatelital permitió la identificación de subgrupos de muestras con MSI-H, MSI-L y MSS, hallazgos que coinciden con los reportados en el cáncer colorrectal esporádico ^15^^,^^19^^,^^22^^,^^30^^,^^40^^,^^41^. La inestabilidad microsatelital se presenta en 15 a 20 % de los casos de cáncer colorrectal esporádico, especialmente por la hipermetilación del promotor del gen *MLH1*; por otra parte, en los pacientes con cáncer colorrectal de tipo hereditario, como el síndrome de Lynch o cáncer colorrectal no polipósico hereditario, la inestabilidad microsatelital se presenta hasta en el 90 % y se origina por mutaciones germinales en los genes del sistema de reparación *Mismatch Repair System*^5^^,^^8^^,^^9^^,^^10^^,^^20^^,^^42^.

En el presente estudio, la inestabilidad microsatelital se detectó con mayor frecuencia en los tumores localizados en el colon ascendente y en el descendente, mientras que no se detectó en el recto, posiblemente por el bajo número de casos. Asimismo, la inestabilidad microsatelital se detectó con mayor frecuencia en cáncer colorrectal de estadios I y II. En general, nuestros resultados concuerdan con los reportados en otros estudios y corroboran la variación de la inestabilidad microsatelital a lo largo del colon hasta el recto, así como su presencia en estadios tempranos de la carcinogénesis del cáncer colorrectal esporádico ^10^^,^^15^^,^^16^^,^^30^^,^^40^^,^^42^.

Por otra parte, en las 12 muestras de cáncer colorrectal positivo para inestabilidad microsatelital, no se detectó la mutación V600E del gen *BRAF*; este resultado difiere de lo reportado en otros estudios en los que se informa que esta es más frecuente en muestras de cáncer colorrectal con MSI-H ^16^^,^^19^^,^^22^^,^^30^^,^^43^^,^^44^. En el presente estudio, cinco de las 12 muestras con inestabilidad microsatelital tenían MSI-H, por lo que el bajo número de muestras podría explicar que no se haya identificado esta mutación. Sin embargo, nuestros resultados concuerdan con los publicados por otros autores que no encontraron esta relación en pacientes con cáncer colorrectal esporádico ^45^^,^^46^. En sentido contrario a la ausencia de mutaciones en el gen BRAF, en este estudio se encontró que el 16 % de los tumores positivos para inestabilidad microsatelital tenía mutaciones en el *KRAS*, siendo la mutación G12D la más frecuente, lo cual coincide con lo reportado por otros estudios ^16^^,^^19^^,^^22^^,^^30^^,^^42^^,^^43^^,^^47^. En general, nuestros hallazgos nos permiten sugerir que, en los casos de cáncer colorrectal esporádico, los positivos para inestabilidad microsatelital constituyen un subgrupo molecular que se caracteriza por presentar una baja frecuencia de mutaciones en los genes *APC*, *KRAS*, *BRAF* y *TP53*; en tanto que los pacientes con estabilidad microsatelital se caracterizan por presentar una mayor frecuencia de mutaciones en los genes analizados. Ello permite concluir que son dos subgrupos con características moleculares diferentes.

Es importante resaltar la utilidad clínica de evaluar la inestabilidad microsatelital en los pacientes con cáncer colorrectal, puesto que en diversos estudios clínicos se ha informado que los pacientes con MSI-H en estadio II tienen un mejor pronóstico de la enfermedad y no se benefician de el tratamiento con 5-fluorouracilo, por lo que se concluye que la inestabilidad microsatelital es un biomarcador predictivo negativo para esta terapia. Además, se ha observado que los pacientes con tumores positivos para inestabilidad microsatelital tienen una mayor tasa de mutación que los tumores con estabilidad microsatelital, lo que podría aumentar el riesgo de desarrollar tumores extracolónicos. ^10^^,^^16^^,^^18^^,^^19^^,^^20^^,^^40^^,^^42^.

En nuestro estudio, se encontró una alta frecuencia de metilación del promotor del gen *MLH1* (73,1 %), lo que concuerda con la informada en otros estudios en cáncer colorrectal esporádico ^19^^,^^22^^,^^27^^,^^48^^-^^50^. Este es el primer reporte en una cohorte de pacientes colombianos con cáncer colorrectal esporádico y dicha frecuencia de metilación está entre las más altas reportadas. El *MLH1* es un gen de reparación del sistema *Mismatch Repair System*, y su inactivación por la hipermetilación en el promotor se ha descrito ampliamente en el cáncer colorrectal y en otros tumores sólidos, por lo que se considera un evento común en el desarrollo del colorrectal ^5^^,^^7^^,^^8^^,^^20^. En este estudio, la metilación del gen *MLH1* fue más común en los tumores localizados en el colon ascendente en estadios I y II, y menos común en el recto, lo que es similar a lo encontrado en los análisis de MSI. En general, nuestros hallazgos coinciden con los informados en la literatura en cuanto a una mayor frecuencia de metilación del *MLH1* en el colon proximal y en estadios tempranos del desarrollo del cáncer colorrectal ^16^^,^^19^^,^^22^^,^^42^^,^^43^^,^^48^. También, corroboran que la metilación del gen MLH1 disminuye en el trayecto del colon proximal hacia el recto, lo que podría sugerir que se presentan diferentes vías carcinogénicas en estos sitios anatómicos del intestino ^15^^,^^16^^,^^19^^,^^22^^,^^27^^,^^49^.

En nuestro estudio, el 91,6 % de las muestras con inestabilidad microsatelital tenían metilación en el gen *MLH1*; este hallazgo concuerda con la relación directa entre la metilación de este gen y la inestabilidad microsatelital en el cáncer colorrectal esporádico reportada en la literatura ^16^^,^^19^^,^^22^^,^^30^^,^^43^^,^^48^^,^^51^. Sin embargo, nuestros resultados también mostraron una alta frecuencia de metilación del promotor del gen *MLH1* en el grupo de tumores con estabilidad microsatelital, lo que podría indicar que la metilación del *MLH1* no es un evento exclusivo que ocurre en los tumores positivos para inestabilidad microsatelital y que, en general, es un fenómeno común que se presenta en la carcinogénesis del cáncer colorrectal.

Dada la relación directa entre la inestabilidad microsatelital y la metilación del gen *MLH1* en el cáncer colorrectal y la presencia de las dos alteraciones en los estadios I y II, se sugiere que se consideren como biomarcadores de gran utilidad clínica para el diagnóstico genético en los estadios iniciales de esta neoplasia.

En el presente estudio, se observó que el grupo de tumores con estabilidad microsatelital y metilación en el gen *MLH1* se caracterizó por presentar una mayor frecuencia de mutaciones en los genes *APC*, *KRAS* y *TP53*, contrario a lo observado en el grupo de tumores con inestabilidad microsatelital. A partir de estos resultados, se concluye que la metilación del gen *MLH1* fue la alteración molecular más común detectada en las muestras analizadas de cáncer colorrectal y que esta, posiblemente, se presenta por una vía de carcinogénesis independiente a la vía de la inestabilidad microsatelital. Además, se sugiere que los pacientes evaluados con cáncer colorrectal presentan subgrupos con características moleculares diferentes.

Por último, el análisis global de los resultados del perfil de mutaciones, la inestabilidad microsatelital y el patrón de metilación del gen *MLH1* en los pacientes con cáncer colorrectal esporádico estudiados, permitió una mejor comprensión de la caracterización molecular de este tipo de cáncer y la identificación de dos subgrupos de pacientes con perfiles genéticos diferentes. El primero se caracterizó por presentar una inestabilidad microsatelital positiva, alta frecuencia de metilación del gen *MLH1* y bajas frecuencias de mutaciones en los genes *APC*, *KRAS*, *BRAF* y *TP53*. El segundo subgrupo se caracterizó por presentar estabilidad microsatelital, alta frecuencia de metilación del *MLH1* y una mayor frecuencia de mutaciones en los genes *APC*, *KRAS* y *TP53*. Estos subgrupos moleculares, identificados y reportados por primera vez en nuestra población, concuerdan con los informados por el consenso de subtipos moleculares del cáncer colorrectal. Lo anterior nos permite concluir que los pacientes con cáncer colorrectal esporádico estudiados presentaron diferentes tipos de alteraciones moleculares en la vía supresora o de inestabilidad cromosómica, microsatelital o epigenética. Los hallazgos obtenidos en nuestro estudio confirman la heterogeneidad molecular descrita en el desarrollo del cáncer colorrectal.

En conclusión, este es el primer estudio en nuestro país de evaluación simultánea de las vías de mutaciones en los genes *APC*, *KRAS*, *TP53* y *BRAF*, de la inestabilidad microsatelital y epigenética en pacientes con cáncer colorrectal, lo que permitió identificar subgrupos moleculares diferentes según las vías alteradas. Además, los pacientes con cáncer colorrectal presentaron un perfil de mutaciones que difiere del de otras poblaciones.
